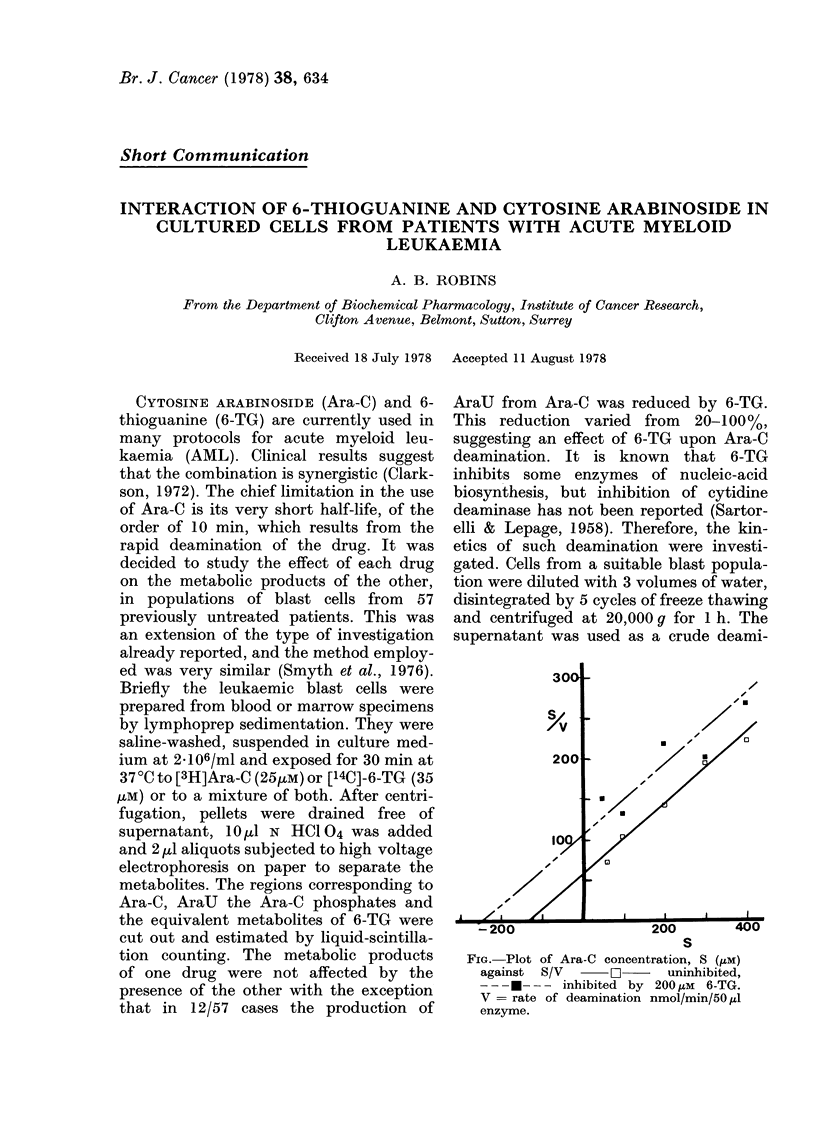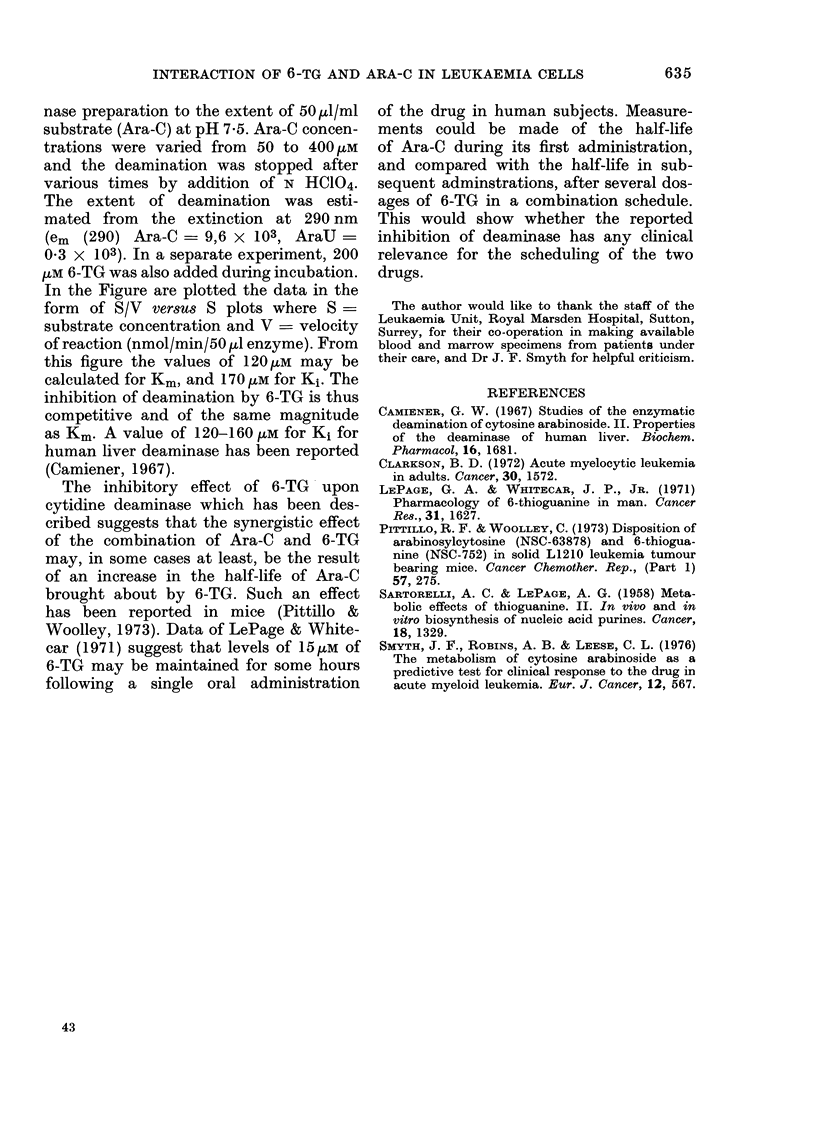# Interaction of 6-thioguanine and cytosine arabinoside in cultured cells from patients with acute myeloid leukaemia.

**DOI:** 10.1038/bjc.1978.261

**Published:** 1978-11

**Authors:** A. B. Robins


*Br. J. Cancer* (1978) **38**, 634

***Short Communication***

### INTERACTION OF 6-THIOGUANINE AND CYTOSINE ARABINOSIDE IN CULTURED CELLS FROM PATIENTS WITH ACUTE MYELOID LEUKAEMIA

A. B. ROBINS

*From the Department of Biochemical Pharmacology, Institute of Cancer Research, Clifton Avenue, Belmont, Sutton, Surrey*

Received 18 July 1978 Accepted 11 August 1978

CYTOSINE ARABINOSIDE (Ara-C) and 6-thioguanine (6-TG) are currently used in many protocols for acute myeloid leukaemia (AML). Clinical results suggest that the combination is synergistic (Clarkson, 1972). The chief limitation in the use of Ara-C is its very short half-life, of the order of 10 min, which results from the rapid deamination of the drug. It was decided to study the effect of each drug on the metabolic products of the other, in populations of blast cells from 57 previously untreated patients. This was an extension of the type of investigation already reported, and the method employed was very similar (Smyth *et al.*, 1976). Briefly the leukaemic blast cells were prepared from blood or marrow specimens by lymphoprep sedimentation. They were saline-washed, suspended in culture medium at $2 \cdot 10^6$/ml and exposed for 30 min at 37 °C to [$^3$H]Ara-C (25 μM) or [$^{14}$C]-6-TG (35 μM) or to a mixture of both. After centrifugation, pellets were drained free of supernatant, 10 μl N $HClO_4$ was added and 2 μl aliquots subjected to high voltage electrophoresis on paper to separate the metabolites. The regions corresponding to Ara-C, AraU the Ara-C phosphates and the equivalent metabolites of 6-TG were cut out and estimated by liquid-scintillation counting. The metabolic products of one drug were not affected by the presence of the other with the exception that in 12/57 cases the production of AraU from Ara-C was reduced by 6-TG. This reduction varied from 20–100%, suggesting an effect of 6-TG upon Ara-C deamination. It is known that 6-TG inhibits some enzymes of nucleic-acid biosynthesis, but inhibition of cytidine deaminase has not been reported (Sartorelli & Lepage, 1958). Therefore, the kinetics of such deamination were investigated. Cells from a suitable blast population were diluted with 3 volumes of water, disintegrated by 5 cycles of freeze thawing and centrifuged at 20,000 *g* for 1 h. The supernatant was used as a crude deami-

FIG.—Plot of Ara-C concentration, S (μM) against S/V ——□—— uninhibited, – – –■– – – inhibited by 200 μM 6-TG. V = rate of deamination nmol/min/50 μl enzyme.

nase preparation to the extent of 50 μl/ml substrate (Ara-C) at pH 7·5. Ara-C concentrations were varied from 50 to 400 μM and the deamination was stopped after various times by addition of N $HClO_4$. The extent of deamination was estimated from the extinction at 290 nm ($e_m$ (290) Ara-C = 9,6 × $10^3$, AraU = 0·3 × $10^3$). In a separate experiment, 200 μM 6-TG was also added during incubation. In the Figure are plotted the data in the form of S/V *versus* S plots where S = substrate concentration and V = velocity of reaction (nmol/min/50 μl enzyme). From this figure the values of 120 μM may be calculated for $K_m$, and 170 μM for $K_i$. The inhibition of deamination by 6-TG is thus competitive and of the same magnitude as $K_m$. A value of 120–160 μM for $K_i$ for human liver deaminase has been reported (Camiener, 1967).

The inhibitory effect of 6-TG upon cytidine deaminase which has been described suggests that the synergistic effect of the combination of Ara-C and 6-TG may, in some cases at least, be the result of an increase in the half-life of Ara-C brought about by 6-TG. Such an effect has been reported in mice (Pittillo & Woolley, 1973). Data of LePage & Whitecar (1971) suggest that levels of 15 μM of 6-TG may be maintained for some hours following a single oral administration of the drug in human subjects. Measurements could be made of the half-life of Ara-C during its first administration, and compared with the half-life in subsequent adminstrations, after several dosages of 6-TG in a combination schedule. This would show whether the reported inhibition of deaminase has any clinical relevance for the scheduling of the two drugs.

The author would like to thank the staff of the Leukaemia Unit, Royal Marsden Hospital, Sutton, Surrey, for their co-operation in making available blood and marrow specimens from patients under their care, and Dr J. F. Smyth for helpful criticism.

### REFERENCES

Camiener, G. W. (1967) Studies of the enzymatic deamination of cytosine arabinoside. II. Properties of the deaminase of human liver. *Biochem. Pharmacol*, **16**, 1681.

Clarkson, B. D. (1972) Acute myelocytic leukemia in adults. *Cancer*, **30**, 1572.

LePage, G. A. & Whitecar, J. P., Jr. (1971) Pharmacology of 6-thioguanine in man. *Cancer Res.*, **31**, 1627.

Pittillo, R. F. & Woolley, C. (1973) Disposition of arabinosylcytosine (NSC-63878) and 6-thioguanine (NSC-752) in solid L1210 leukemia tumour bearing mice. *Cancer Chemother. Rep.*, (Part 1) **57**, 275.

Sartorelli, A. C. & LePage, A. G. (1958) Metabolic effects of thioguanine. II. *In vivo* and *in vitro* biosynthesis of nucleic acid purines. *Cancer*, **18**, 1329.

Smyth, J. F., Robins, A. B. & Leese, C. L. (1976) The metabolism of cytosine arabinoside as a predictive test for clinical response to the drug in acute myeloid leukemia. *Eur. J. Cancer*, **12**, 567.